# The importance of membrane microdomains for bile salt-dependent biliary lipid secretion

**DOI:** 10.1242/jcs.211524

**Published:** 2018-03-01

**Authors:** Johannes Eckstein, Hermann-Georg Holzhütter, Nikolaus Berndt

**Affiliations:** Charité – Universitätsmedizin Berlin, Institute of Biochemistry, Charitéplatz 1, 10117 Berlin, Germany

**Keywords:** Bile formation, Bile salts, Mathematical modeling, Membrane lipid, Microdomain

## Abstract

Alternative models explaining the biliary lipid secretion at the canalicular membrane of hepatocytes exist: successive lipid extraction by preformed bile salt micelles, or budding of membrane fragments with formation of mixed micelles. To test the feasibility of the latter mechanism, we developed a mathematical model that describes the formation of lipid microdomains in the canalicular membrane. Bile salt monomers intercalate into the external hemileaflet of the canalicular membrane, to form a rim to liquid disordered domain patches that then pinch off to form nanometer-scale mixed micelles. Model simulations perfectly recapitulate the measured dependence of bile salt-dependent biliary lipid extraction rates upon modulation of the membrane cholesterol (lack or overexpression of the cholesterol transporter Abcg5–Abcg8) and phosphatidylcholine (lack of Mdr2, also known as Abcb4) content. The model reveals a strong dependence of the biliary secretion rate on the protein density of the membrane. Taken together, the proposed model is consistent with crucial experimental findings in the field and provides a consistent explanation of the central molecular processes involved in bile formation.

## Abstract (precis)

**Summary:** Mathematical modeling of canalicular membrane domain formation reconciles a membrane extraction mechanism for biliary lipid secretion with putatively opposing experimental findings.

## INTRODUCTION

Production of the bile is an important liver function required for the efficient digestion of dietary lipids and the elimination of xenobiotics and endogenous waste products. In the process of bile formation, bile salts (BSs) are actively pumped from the cytosol of hepatocytes into the extracellular space where they act as detergents solubilizing phospholipids, cholesterol and proteins from the outer leaflet of the canalicular membrane.

The molecular mechanism by which the central lipids of the bile, phosphatidylcholine (PC) and cholesterol (CH), are secreted from the canalicular membrane into the extracellular space and packed together with BSs in mixed micelles or vesicles is still a matter of debate. The extraction model (E-model) assumes that BS micelles formed in the canalicular space successively extract PC and CH molecules from the outer leaflet. Initially, PC is the preferentially extracted lipid thereby increasing the CH-solubilizing capacity. Later, PC, CH and BS mixed micelles are formed (Small, 2003). In this model, the secretion rate of individual lipids into the bile is independent from each other and mainly determined by the activity of ABC flippases transporting PC and CH from the inner to the outer leaflet. The E-model is consistent with several experimental findings on changes in the lipid composition of the bile of transgenic animals lacking one or two of the ABC transporters. Disruption of the gene for the lipid transporter Mdr2 (also known as Abcb4) in mice, which is responsible for the ‘flopping’ of PC from the inner to the outer membrane leaflet, was accompanied by a complete absence of phospholipid from the bile (Smit et al., 1993). Similarly, knockdown of the CH transporter Abcg5–Abcg8 in mice reduced the secretion rate of CH by 70–90% (Kosters et al., 2006; Yu et al., 2002a).

Importantly, however, there was no significant change of the CH secretion rate in Abcg8^−/−^ mice that have an altered lipid composition of the canalicular membrane due to the simultaneous knockdown of the serine flippase Atp8b1 (Groen et al., 2008). This finding suggested that mechanisms other than the Abcg5–Abcg8-mediated loading of CH on BS micelles are involved in the secretion of CH. One possible alternative mechanism is the BS-dependent budding of mixed micelles from the canalicular membrane. This budding model (B-model) assumes that budding of membrane patches is a central mechanism of bile micelle formation instead of extraction of single lipids from the membrane by preformed BS micelles as assumed in the E-model. This model relies on electron microscopy studies (Crawford et al., 1995) and mathematical modeling (Eckstein et al., 2015; Marrink and Mark, 2002). According to the B-model, the lipid composition of the bile should reflect the lipid composition of membrane region from which bile micelles are being released. Experimental studies suggest that BS-dependent extraction of membrane constituents takes place preferentially from liquid disordered (Ld) microdomains that are enriched in the membrane lipid PC (Tietz et al., 2005). Hence, interpreting experimental data on biliary secretion rate on the basis of the B-model requires knowledge of the size distribution and lipid composition of Ld microdomains. While the chemical composition of the outer leaflet as a whole can be assessed by various experimental techniques [chemical labeling with non-penetrating agents, immunological methods, phospholipase digestion of membrane phospholipids, use of phospholipid-exchange proteins, and physicochemical methods such as X-ray diffraction and NMR (Tietz et al., 2005)] the distribution of lipids and proteins across liquid ordered (Lo) and Ld microdomains can only be indirectly inferred from the chemical composition of detergent-resistant membranes (DRMs) (Mazzone et al., 2006). Whether this method truly informs about the composition of microdomains in the intact membrane under *in vivo* conditions is highly questionable (Lichtenberg et al., 2005).

We thus strived in this work to construct a reliable mathematical model of biliary lipid secretion that picks up the concept of the B-model. To this end, we first extended our previous mathematical model of microdomain formation (Eckstein et al., 2015) by including lipid–protein interactions. This was necessary because our previous model had some limitations in correctly describing the size distribution and lifetime of microdomains because only lipid–lipid interactions were considered. In this case, membrane lipids progressively de-mixed and eventually segregated into single large Lo and Ld ‘macrodomains’. Formation of such stable macro-scale domains is indeed observed in artificial protein-free membrane systems (AMSs) (Kahya et al., 2003), but not in biological membranes. Hence, to circumvent the problem of complete lipid de-mixing, we had to introduce in our previous model an average domain life time at which the simulation of domain growth was interrupted owing to the virtual extraction of a membrane patch. To get rid of this obvious limitation, we extended our model by including protein–lipid interactions as a possible mechanism that may constrain the continuous growth of domains and enable the formation of time-stable microdomains.

We further combined our extended model of microdomain formation with a model of micelle budding from Ld domains that is in concordance with biophysical principles governing the solubilization of biological membranes by detergents (Helenius and Simons, 1975; Lichtenberg et al., 2013). According to this model, BS monomers preferentially intercalate into Ld domains and facilitate the budding of nanometer-scale mixed micelles. We demonstrate that the model can perfectly recapitulate experimentally observed variations in the lipid composition of the bile and BS-dependent secretion rates under normal conditions as well as at altered activity of the ABC transporters for PC and CH. We discuss several other aspects substantiating the feasibility the membrane budding concept as the dominating mechanism of biliary lipid secretion.

## RESULTS

### Model description

#### Lattice model

The modeling approach developed in our preceding work (Eckstein et al., 2015) was extended by including membrane proteins and their interaction with membrane lipids. In brief, the model represents the outer membrane leaflet as a triangular lattice of 122×122 cells with 1 nm^2^ of cell size corresponding to an upper boundary of cross-sectional area occupied by a typical membrane lipid. Each lattice cell is occupied either by a lipid or a protein unit. We distinguish between three basic types of membrane lipids: CH, phosphatidylcholine (PC) and sphingomyelin (SM). They represent the prevailing membrane lipids in the outer leaflet of the canalicular membrane, and serve as prototypes for other membrane lipids with similar chemical properties.

Proteins are represented as hexagonal-shaped non-deformable clusters of ‘units’ occupying 19, 37 or 61 lattice cells of the lattice. According to the ‘lipid annulus’ concept (Contreras et al., 2011) the surface of integral membrane proteins is covered with tightly bound ‘rim lipids’ acting like a lubricating layer. The interaction energies of rim lipids with adjacent mobile lipids determine the energetic state of the protein and thus the preference of the protein for the Lo or Ld phase, respectively.

#### Interaction energies and phase-ordering energies

Movement of lipids and proteins is driven by minimization of the total energy *E* of the system according to:
(1)
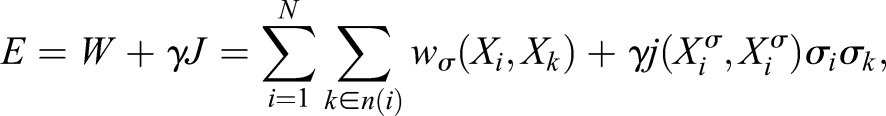
where *X*=CH, PC or SM and specifies the lipid type, the scaling factor *γ* weights the relative contribution of the phase-ordering energy *J* and the interaction energy *W* to the total energy *E*. The index *σ* designates the ordering state of the lipid: *σ*=− 1 denotes the low-ordered state where the bulky conformation of the fatty acid chains allows high flexibility and thus rapid movement of the lipid; *σ*=+ 1 denotes the high-ordered state where the fatty acid chains are stretched and tightly packed thus restricting lipid mobility.

The set of six cells adjacent to cell *i* is denoted by *n*(*i*). *w*_*σ*_(*X*_*i*_, *X*_*k*_) is the matrix of pairwise lipid interactions. There are two such matrices for each ordering state *σ*=± 1. The elements 
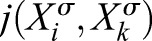
 of the matrix *J* quantify the strength with which a lipid with ordering state *σ* ‘enslaves’ the neighboring lipid to adopt the same ordering state. Numerical values for the elements of the matrices *w*_*σ*_(*X*_*i*_, *X*_*k*_) and 
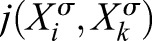
 were taken from our previous work (Eckstein et al., 2015).

Minimization of the total lattice energy *E* was carried out by using the Gillespie algorithm with periodic boundary conditions. For more technical details of the minimization procedure see Eckstein et al. (2015).

#### Particle movement

The movement of mobile lipids is restricted to the pairwise exchange between neighboring cells *i*, *j* with the exchange rate:
(2)
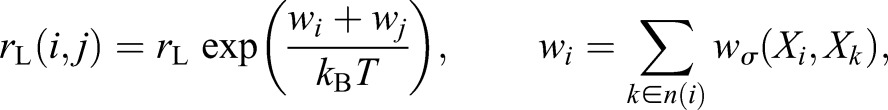
*w*_*i*_ is the total energy of the lipid at lattice position *i* and *r*_L_ is a scaling factor used to relate the time scale of the lattice simulation to the time scale of experiments.

Movement of a protein is modeled as a series of consecutive shifts of protein units to adjacent lattice cells. We implemented two variants for the re-distribution of the lipids displaced by the moving front of the protein: (1) shift to lattice cells that prior to the move are occupied by the protein units constituting the back of the protein, (2) successive sequential displacement of lipids along the surface of the protein like a bow wave. Both variants yielded identical simulation results. The rate of protein movement is calculated from Eqn 3:
(3)
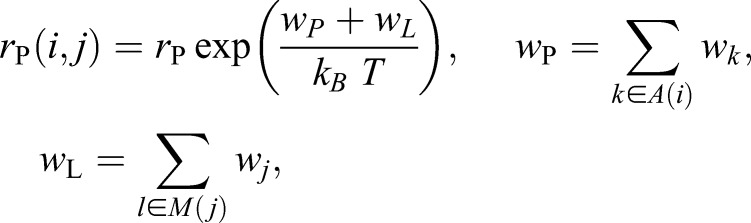
where *w*_P_ denotes the energy of the protein at position *i*, with *A*(*i*) the set of cells defining its rim. *w*_L_ is the total energy of all *M*(*j*) lipids moved by the movement of the protein in the direction of lattice cell *j*. The scaling factor *r*_P_ relates the time scales of protein to those of the lipid movement.

In our model, the plethora of different membrane proteins is represented by two generic proteins, which we will refer to in the following as raft protein (RP) and non-raft protein (NRP) owing to their preferences for the Lo and Ld phase, respectively. This lipid preference is controlled by the interaction energies of the rim lipids *X*_P_ of the protein with the adjacent mobile lipids *X*_*k*_,
(4)
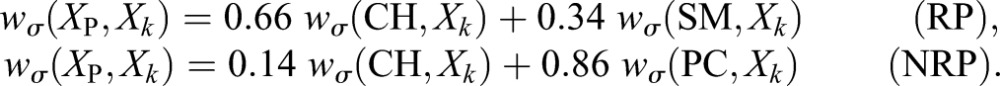
The coefficients in Eqn 4 equal the fraction of CH and SM in the Lo phase and of CH and PC in the Ld phase, respectively.

#### Diffusion and mobility

The ‘diffusivity’ of a particle (lipid, protein) is expressed through the diffusion coefficient *D* that up to a scaling factor relates to the mean squared displacement Δ*x*(*t*)^2^=4*Dt* where Δ*x*(*t*) is the distance that the particle travels during the time span *t*. The diffusivity of a particle has to be distinguished from its ‘mobility’ which in our model refers to the number of elementary moves accomplished within time *t*. A particle may have a large mobility but a small diffusivity if boundaries exist restricting the possible directionality of the movement, an effect called tortuosity.

#### Definition of the biliary lipid extraction rate

Bile salts are specific organic detergents. Therefore, their interactions with membranes obey the biophysical principles of detergent-dependent membrane solubilization. The general consensus is a three-stage model originally proposed by Helenius and Simons (Helenius and Simons, 1975) comprising: (1) partitioning of detergent between lipid bilayers and the aqueous media, described by a partition coefficient *K*, (2) formation of unstable membrane patches rimmed by detergent BS, and (3) release of these unstable membrane patches in the aqueous phase under formation of mixed micelles. According to this scheme, our model assumes that BSs intercalate into the Ld domains, with the hydrophilic part remaining at the surface and facilitating membrane budding and release of nascent micelles. We defined a membrane patch of size *N* at position *i* as being extractable if it is fully encircled by lipids belonging to the Ld phase:

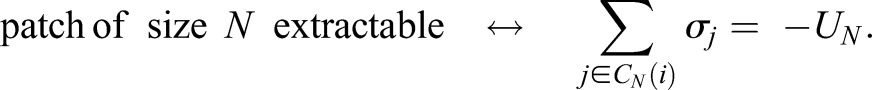
The sum counts the ordering states of the lipids at the rim *C*_*N*_(*i*) of the patch. *U*_*N*_ denotes the length of the rim. In our lattice model it holds that:

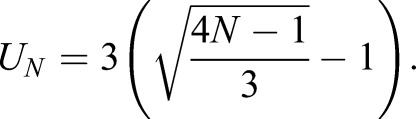


Numerous experimental studies have demonstrated that the solubilized membrane fraction increases with increasing concentration of detergents. We thus made the plausible assumption that the patch extraction rate is proportional to the BS concentration within the patch. Furthermore, it has been shown that detergents like BSs or polymers can arrange into pore-like membrane structures facilitating the excision of the enclosed membrane fragment (Mazer et al., 1984; Schubert and Schmidt, 1988). We thus further postulated that the extraction of a membrane patch may occur only if the number of intercalated BSs is large enough to form a rim of BS molecules around the patch (see also Fig. 7). Based on these assumptions we define the rate of the *i*-th hexagonal membrane patch from the set of all 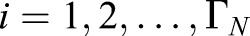 extractable patches with size *N* being extracted from the model membrane as follows:
(5)
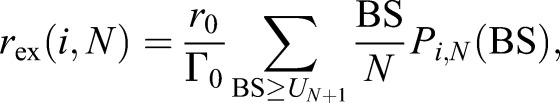
where *r*_0_ is a scaling factor that is used to relate model-based lipid extraction rates to *in vivo* measurements, 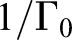 is the probability for randomly choosing a specific patch from the whole set of 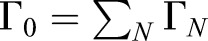 of possible patches and *P*_*i*,*N*_(BS) is the probability (fraction) of BSs in the patch. The factor (BS/*N*) relates the extraction rate linearly to the BS density. The summation term in Eqn 5 runs over BS numbers equal or larger than *U*_*N*+1_ (i.e. large enough to form a ring around the patch of size *N*).

The reversible exchange of BSs between the aqueous phase and the patch we describe by the master equation:
(6)
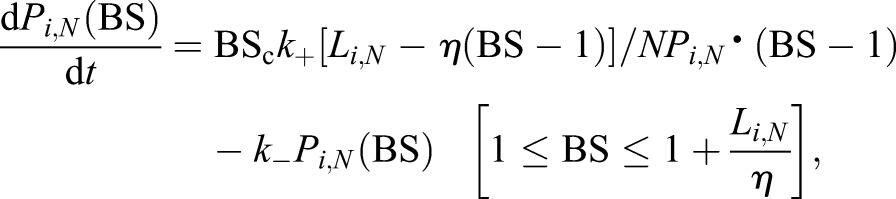
where *k*_+_ and *k*_−_ are the on and off rate constants for the uptake and release of a single BS molecule. *L*_*i*,*N*_ is the number of lipid molecules in the patch and BS_C_ is the number of BS molecules in the canalicular space per membrane unit. The parameter *η* is a solubility index that represents the number of lipid molecules that are required to accommodate one BS molecule. The larger *η*, the smaller is the membrane solubility of a BS.

Assuming a fast equilibrium {i.e. [d*P*_*i*,*N*_(BS)/d*t*]≈0}, Eqn 6 reduces to a recursive algebraic equation the solution of which yields an explicit expression for *P*_*i*,*N*_(BS):
(7)
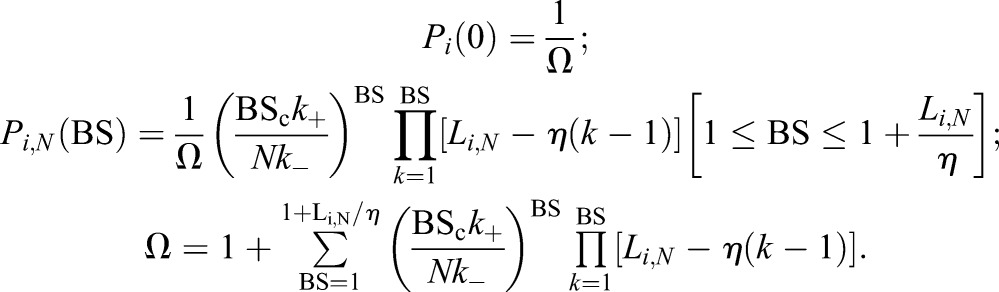
The ratio *K*_BS_=*k*_+_/*k*_−_ of the forward and backward rate constants in Eqn 7 defines the partition coefficient of the BS.

Using Eqn 7 in Eqn 5 yields an explicit expression for the extraction rate of a single patch. To examine the size distribution of extracted patches, we sum up Eqn 5 over all extractable patches of the same size *N* to obtain the mean extraction rate of patches with size *N*:
(8)
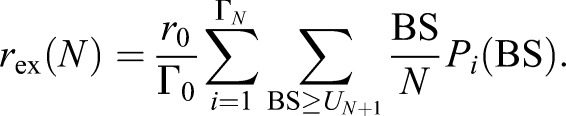
The secretion rate of any membrane species *X* (lipid or protein) associated with the extraction of patches with size *N* is:
(9)
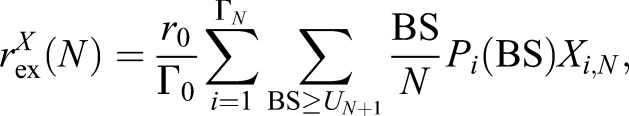
where *X*_*i*,*N*_ is the number of molecules of species *X* contained in the patch. Summing up Eqn 9 over all patch sizes *N* the mean secretion rate of an arbitrary membrane species *X* finally reads:
(10)
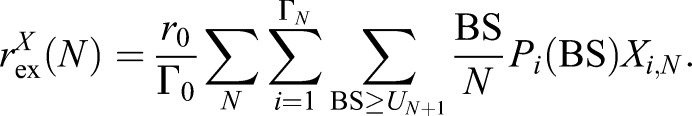


### Comparing simulation results with published data

#### Diffusion of membrane proteins

**Fig. 1.**
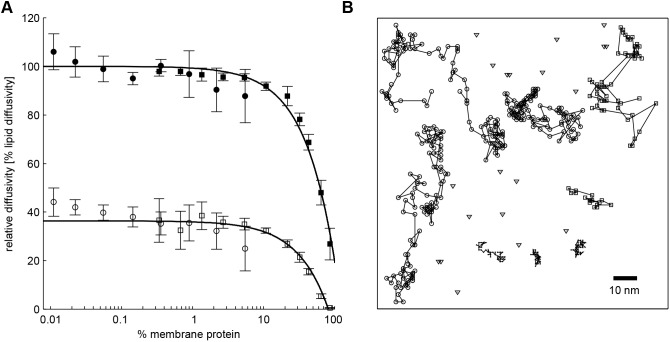
**Simulated diffusion** **of lipids and proteins.** (A) Impact of protein density on the diffusion coefficient of a NRP imbedded into the Ld phase. Circles designate experimental data taken from Ramadurai et al. (2009): empty circles, dimeric lactose transporter (*R*=3 nm); filled circles, lipids [75% dioleoylglycerophosphocholine (DOPC), 25% dioleoylphosphatidylglycerol (DOPG)]. Squares designate simulated data: empty squares, NRPs comprising *N*=37 units (equal to the lattice cells); filled squares, lipids (14% CH and 86% PC ensuring adoption of a monophasic Ld phase). In order to make both systems comparable the diffusion coefficients are shown normalized to the diffusion coefficient of the protein-free lipid system. Very low protein densities (<0.1%) correspond to less than one protein on the lattice and thus could not be simulated. Conversely, high protein densities (>10%) were not accessible to the experiment. (B) Trajectories of selected NRPs and RPs monitored in time steps of 10 µs over a time interval of 1 ms in the steady state of a simulation of the canalicular membrane. Circles, NRPs moving in the Lo phase; dots, NRPs moving in the Lo phase; squares, RPs moving in the Ld phase; triangles, RPs moving in the Lo phase.

First, we fixed the scaling factor *r*_P_ in Eqn 3 such that the simulated diffusion coefficients of membrane proteins were in the expected range. Putting *r*_P_/*r*_L_ to 0.38 and 0.41 for the NRP and RP, respectively, the model simulations correctly recapitulated the measured relationship between the diffusivity of three different non-raft proteins and their membrane densities (Fig. 1A). The overall diffusion coefficients obtained as time averages across the diffusion coefficients for both types of domains were 0.8 µm^2^/s for the NRPs and 0.07 µm^2^/s for the RPs. These values are in the range reported in several experimental studies (Dietrich et al., 2002; Kenworthy et al., 2004; Owen et al., 2009). The simulations uncovered large differences in the protein mobilities in the Ld and Lo domain (see typical trajectories of the RP and NRP in Fig. 1B). Both the RPs and NRPs have much lower mobilities in Lo domains compared to what was found for Ld domains. This is a consequence of the low lipid mobility in Lo domains restricting the displacement of the lipid shell in front of the moving protein (equal to the motion in a viscous/crystalline environment). During random transits from one domain type to the other, the proteins switch between low- and high-mobility regimes.

#### Influence of membrane proteins on domain formation

We used our model to study how the presence of membrane proteins influences the formation of domains in the canalicular membrane of hepatocytes. In these simulations, we probed the effect of variations in the protein density (the percentage of the membrane fraction occupied by proteins) and the protein mobility. The latter aspect is important as membrane proteins can be mobile (free diffusion or energy-dependent transport) or virtually immobile due to anchoring to the cytoskeleton (Goiko et al., 2016; Kikuchi et al., 2002).

The simulations were started with a random distribution of lipids and proteins. The RP:NRP ratio was 47:53 corresponding to the ratio of membrane areas covered by Lo and Ld domains in the protein-free case. This ratio is only slightly higher than the estimated share of ∼33% of total membrane protein resident in lipid rafts purified from hepatocyte plasma membranes in a non-detergent affinity chromatography method (Atshaves et al., 2007).

**Fig. 2.**
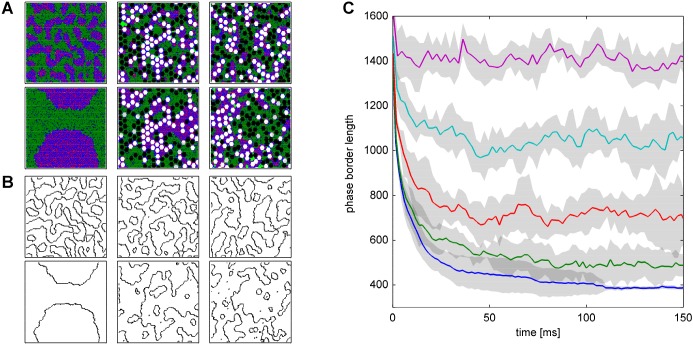
**Domain formation in the apical membrane of hepatocytes.** The lipid composition of the model membrane was SM:CH:PC=15.7:37.8:46.5; protein composition was NRP:RP=53:47; and total protein:total lipid=0.4:0.6. The size of the model protein was *N*=37 nm^2^ corresponding to a protein radius of *R*≈3 nm. (A) Domain structures at different time points (upper row: 0.1 ms, lower row: 125 ms) for a protein-free leaflet (left column), randomly distributed immobile proteins (middle) and diffusing proteins (right column). (B) Phase borders between the Lo and Ld domains shown in A. The total length of the phase borders correlates inversely with the average domain size. (C) Time evolution of the total length of the phase borders used as measures of domain sizes. Proteins are diffusive. Protein densities are changed in steps of 10% from 0% (bottom) to 40% (top). Gray-shaded areas indicate the standard deviation of the stochastic simulations.

In the protein-free membrane, domains progressively grew and collapsed finally into two completely segregated ‘macrodomains’. In contrast, the presence of membrane proteins resulted in the formation of numerous small domains, irrespective of whether the proteins were mobile or immobile (Fig. 2A,B). Simulated domain patterns in the presence of mobile or immobile proteins yielded identical mean domain sizes. However, the temporal and spatial stability of the domains were substantially different. Domains forming in the presence of immobile proteins were stable, retained the spatial location determined by the initial distribution of proteins and displayed only fluctuations of their phase borders. In contrast, domains formed in the presence of mobile proteins continuously changed their spatial position and shape, and were occasionally completely disintegrated and replaced by newly formed domains. It is worthwhile to note, that the domain patterns do not fit a conventional ‘islands in the sea’ view [e.g. as concluded in Meder et al. (2006) from long-range diffusion of model proteins] but rather resemble a maze with ruffled border lines with both phases being continuous (percolating).

We used the total length of the phase borders separating Lo and Ld domains from each other to quantify the average domain size (see Fig. 2C). Constancy of its mean over a time interval of 50 ms was used as criterion for the accomplishment of a stationary domain size distribution.

#### BS-dependent lipid secretion rates of wild-type mice

**Fig. 3.**
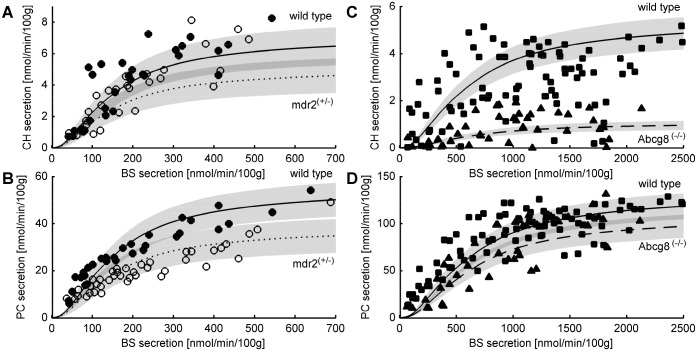
**Taurocholate-dependent biliary secretion of PC and CH.** (A,B) Secretion rates of PC and of CH for wild-type mice (filled circles) and the heterozygous Mdr2^+/−^ mice (open circles). Experimental data were taken from Oude Elferink et al. (1995). The solid line represents the calculated mean secretion rates for the wild-type mice (PC content of the outer leaflet=47%). The dotted line represents the calculated mean secretion rates for the Mdr2^+/−^ mice (PC content of the outer leaflet lowered to 37%). Shaded areas mark the range of computed secretion rates obtained from six recurrent independent stochastic model simulations. The model parameters were: *K*_BS_=5 · 10^−4^ and *η*=1, lipid compositions are 38% CH, 47% PC, 15% SM (wild type) and 45% CH, 37% PC, 18% SM (Mdr2^+/−^). (C,D) Secretion rates of PC and of CH for wild-type mice (squares) and the homozygous Abcg8^−/−^ mice (triangles). Experimental data were taken from Groen et al. (2008). The solid line represents the calculated mean secretion rates for the wild-type mice (CH content of the outer leaflet=25%). The dashed line represents the calculated mean secretion rates for the Abcg8^−/−^ mice (CH content of the outer leaflet lowered to 13%). Shaded areas mark the range of computed secretion rates obtained from 12 recurrent independent stochastic model simulations. The model parameters were: *K*_BS_=1 · 10^−4^ and *η*=1, lipid compositions are 24% CH, 47% PC, 29% SM (wild type) and 14% CH, 47% PC, 40% SM (Abcg8^−/−^).

The simulations outlined in this section were carried out for mobile proteins for which (in contrast to immobile proteins) the domain patterning is an emergent property that is not predefined by the choice of the initial protein distribution. As above, the reference case was defined by a protein density of 40%, a ratio NRP:RP=53:47 and a lipid composition of SM:CH:PC=15.7:37.8:46.5. Based on Eqns 8–10 we calculated the extraction rate of membrane patches and membrane components (lipids, proteins) for various concentrations of BS. The simulations were compared with data from two independent experiments in which the taurocholate (TC)-dependent biliary lipid secretion rates of PC and CH were measured in wild-type mice and in mice either affected by a Mdr2 gene disruption (Oude Elferink et al., 1995) (Fig. 3A,B) or carrying a genetic defect in the CH transporter ABCG5–ABCG8 (Groen et al., 2008) (Fig. 3C,D). As the absolute lipid secretion rates, half-saturation concentrations of TC and the biliary CH:PC ratio differed in these two experiments, we used different values for the model parameter *K*_BS_=*k*_+_/*k*_−_ (membrane/water partition coefficient) and slightly differing lipid compositions of the membrane to fit the wild-type experiments depicted in Fig. 3.

With increasing BS concentrations, the lipid secretion rates of PC and CH tend towards upper limit values. This saturation characteristic is well reproduced by the model. The half-maximal secretion rates for both PC and CH are obtained at BS secretion rates of ∼150 nmol/min/100 g and ∼600 nmol/min/100 g. Differences in these values are possibly due to differences in the design of the two experiments (see Discussion). According to our secretion model, the existence of a maximum for biliary lipid secretion reflects the saturation of the membrane with BS because the solvation of each BS molecule in the membrane ‘consumes’ *η* lipid molecules so that the secretion rate of an individual membrane patch cannot be further elevated if all lipids are consumed.

As shown in Table 1, over the wide range of BS secretion rates considered in the model simulations, the lipid composition of the normal bile shows a remarkable constancy. PC, CH and SM are extracted relative to each other in the ratio of about 88:11:1, in good agreement with reported experimental values (≈85%, 15%, <1% (Nibbering et al., 2001).

**Table 1.**
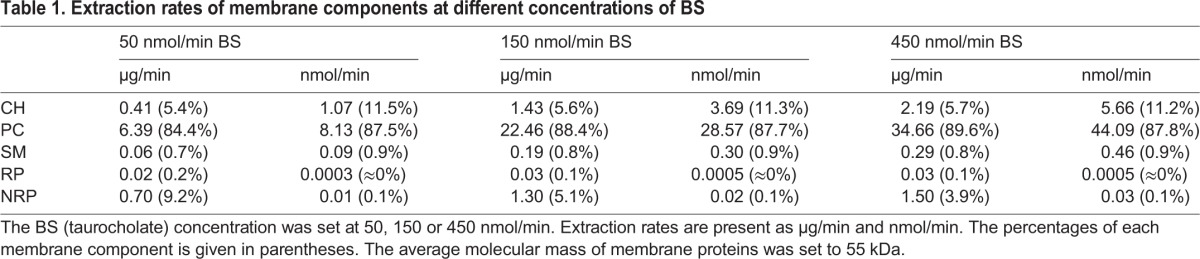
**Extraction** **rates of membrane components at different concentrations of BS**

#### BS-dependent lipid secretion rates of mice defective in lipid transporters

MDR3 (Mdr2 in mice) is an ATP-dependent transporter of amphipathic cationic and neutral compounds implicated in the transmembrane transport of PC from the inner to the outer leaflet (Smit et al., 1993). Hence, genetic ablation of this transporter should lead to a decrease of the PC content in the outer leaflet. We used the model to estimate the reduction of the PC content in the outer leaflet that might account for the reduced PC and CH secretion rates observed in mice heterozygous or homozygous for an Mdr2 gene disruption. To this end, we varied the PC content of the membrane from 0% to 50% while fixing the CH:SM ratio to 71:29, the protein density to 40% and putting the RP:NRP ratio equal to the size ratio of the membrane fraction occupied by Lo and Ld domains in the protein-free membrane. The latter setting was made to take into account that PC depletion of the membrane reduces the size and relative fraction of Ld domains thereby reducing the membrane area available for the accommodation of NRP. The best concordance with the measured PC and CH output rates for the Mdr2^+/−^ mice was obtained by lowering the PC content of the outer leaflet from the initial 47% to 37% for the heterozygous mice (Fig. 3B). This result suggests that an even modest reduction of the membrane PC content by about 20% was sufficient to account for the observed 30% drop of the maximal PC secretion rate of the Mdr2^+/−^ mice. Notably, with the estimated reduction of PC to 37%, the model also correctly recapitulated the drop of the maximal CH secretion rate to 70% of Mdr2^+/−^ mice.

**Fig. 4.**
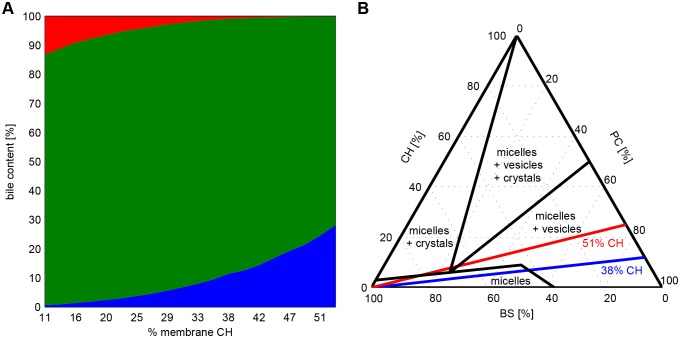
**Impact of membrane CH on biliary CH content and formation of bile crystals.** (A) Computed lipid composition of the bile (red, SM; green, PC; blue, CH) as function of the CH content of the membrane. The sum of CH and SM in the membrane was kept at 53.5%. (B) Equilibrium CH–BS–phospholipid ternary phase diagram for the BS taurocholate adopted from Portincasa et al. (2002). The blue and red line represent the computed BS-dependent biliary content of PC, CH at a membrane CH content of 38% (normal case) and 51% (cholesterol hyper saturation).

We also studied how the CH content of the outer leaflet may influence BS-dependent lipid secretion rates. Membrane depletion of CH may result from an impaired delivery of CH to the membrane or a genetic defect of the CH transporter Abcg5–Abcg8 (Groen et al., 2008). In these computations, we kept the PC content of the outer leaflet constant. This is equivalent to compensating for the drop of CH with a corresponding increase of SM. Remarkably, this setting did not change the relative abundance and size distribution of microdomains and hence preserved the integrity of the membrane. This model finding is in concordance with the observation that no obvious physical differences were apparent between the Abcg5–Abcg8-deficient mice and wild-type mice (Yu et al., 2002a). The domain-structure-preserving effect achieved by replacing CH with SM is due to the fact that SM alone is capable of creating a stable Lo phase. This model-based finding has also been demonstrated experimentally (Frisz et al., 2013; Wang and Silvius, 2003). As shown in Fig. 4A, a decrease of the CH content of the membrane was paralleled by a pronounced decrease of the CH secretion rate, whereas the PC secretion rate remained virtually unaffected. A good concordance between the simulated and measured BS dependence of the CH secretion rate was obtained by reducing the membrane CH content from an initial 38% to 25%. Such a reduction of the membrane content of the Abcg5–Abcg8-deficient mice was indeed reported in Kosters et al. (2006).

We also investigated the effect of a pathologically high CH content in the outer leaflet on biliary CH secretion. Varying the membrane content of CH in a broad range between 11% and 51% at a typical BS output of 600 nmol/min/100 g yielded a stable coexistence of Lo and Ld microdomains. The relative CH content of the bile increased in a hyper-linear fashion up to 25% at a membrane CH content of 51% (see Fig. 4A). Such a fivefold increased CH content of the bile is in good agreement with reported values for the Abcg5–Abcg8-overexpressing mice (Yu et al., 2002b). At high CH loading of the membrane, the model predicts a critical range of the biliary BS content (at ∼70%) at which the lipid composition of mixed micelles may even favor the formation of bile crystals (see red curve in Fig. 4B). This model prediction is consistent with the observation in Yu et al. (2002b) showing that the bile from transgenic animals was opaque and had large amounts of amorphous material as determined by microscopic inspection.

#### Size distribution and lipid composition of extracted membrane patches

**Fig. 5.**
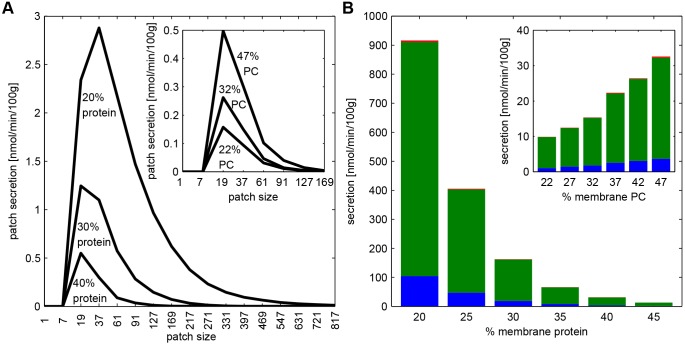
**Impact of membrane protein and PC on patch extraction and secretion rates.** (A) Size distribution of extracted membrane patches for membrane protein densities of 40%, 30% and 20% (membrane PC content: 47%) and different membrane PC content (inset) of 47%, 32% and 22% (membrane protein density: 40%). Values are calculated at the half-maximal BS secretion of 150 nmol/min/100 g with parameters K_BS_=5 · 10^−4^ and *η*=1. (B) The secretion rate of PC (green), CH (blue), SM (red) and proteins (black) are shown for different membrane protein densities (membrane PC content: 47%) and different membrane PC content (membrane protein density: 40%). Values are calculated at the half-maximal BS secretion of 150 nmol/min/100 g with parameters K_BS_=5 · 10^−4^ and *η*=1.

As shown in Fig. 5A, our model predicts a rather narrow size distribution of secreted membrane patches (which are nascent mixed micelles) around the peak value at *N*=20 lipids. The absence of larger patches (*N*>100) reflects the size distribution of Ld domains, which on average have a size of about 35 lipids. It has to be noted that the predicted size range of extracted patches (≈20–60 lipids) is below the size range of 100–400 lipids reported for primary PC micelles (Hofmann and Small, 1967; Small et al., 1969) and for micelles in the native bile from the dog (Mazer et al., 1984). This discrepancy suggests that the extracted nascent micelles are unstable and rapidly aggregate to larger stable micelles (Nichols and Ozarowski, 1990). Notably, despite the large impact of variations in the protein density and PC content on absolute extraction rates of patches, there is little impact on the relative composition of the bile (Fig. 5B).

#### Biliary lipid secretion at various protein density and for different BS species

**Fig. 6.**
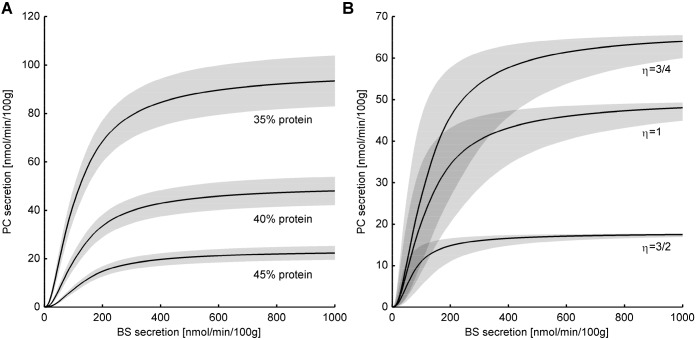
**Impact of membrane protein content and the membrane solubility of BS on lipid secretion into the bile.** (A) Secretion rates of PC at normal lipid composition of the membrane and three different protein densities. The middle line refers to a normal density of 40%, the upper and lower line refer to a reduced (35%) and increased (45%) density of membrane proteins. Shaded areas mark the range of computed secretion rates obtained from 12 recurrent independent stochastic model simulations. The parameters used were K_BS_=5 · 10^−4^ and *η*=1. (B) Secretion rates of PC at normal lipid composition of the membrane but altered model parameters for the interaction of BS with the membrane: η=1.5 (low membrane solubility of the BS), the middle to η=1.0 (normal solubility), η=0.75 (high solubility). The solid lines were computed by setting the value of the partition coefficient as K_BS_=5 · 10^−4^. The shaded areas show the influence of varying K_BS_; the lower margin of the shaded areas refer to K_BS_=2.5 · 10^−4^, the upper margins refer to K_BS_=1 · 10^−3^.

Finally, we studied the possible impact of changing protein concentrations and membrane solubility of BS on the lipid extraction rate. As expected from the strong influence of the protein density on the size of Ld domains (see Fig. 2A), even moderate changes of the protein density from 40% to 35% or 45%, respectively, gave rise to a change of lipid secretion rates for PC and CH by a factor of about two (Fig. 6A). Intriguingly, an increase of the protein content to 45% lowers the lipid secretion rate to the same extent as a decrease of the PC content to 32%, which is below the value associated with the lowered capacity of the heterozygous MDR2 (37%). Note that in these simulations (changing the protein content of the external leaflet and the physico-chemical properties of the BS), we have made the assumption that the predicted increase of the maximal secretion rate can be fully counter-balanced by the rate of membrane replenishment.

In our model, increasing membrane solubility of the BSs is represented by decreasing values of the solubility index *η* determining the number of PC molecules required to adopt one BS molecule. Changing this parameter from 1.5 to 0.75 results in an about fourfold increase in the maximal lipid secretion rate (Fig. 6B). We conclude that elevating the production of BS with high membrane solubility in PC-rich Ld microdomains could be a convenient means to maintain an almost normal lipid secretion rate in cases where the export of otherwise physiologically abundant BS is hampered, for example, due to a genetic defect in the bile salt export pump. For example, secretion of an unexpectedly large amount of tetra-hydroxylated bile acids acid was observed in mice carrying a mutated bile salt pump. The total bile salt output in these mutant mice was still ∼30% of wild type although the secretion of cholic acid was greatly reduced to 6% (Wang et al., 2001). From this observation, one might conclude that the solubility index *η* correlates inversely with the hydrophilicity of the BS. On the other hand, the effect of five different BSs on bile flow revealed a higher membrane-solubilizing potency for hydrophobic BS (Loria et al., 1989). Finally, other studies have failed to establish a significant relationship between bile flow and BS hydrophobicity (Gurantz and Hofmann, 1984). Hence, there is apparently no simple relationship between the hydrophobicity of BS and the solubility index *η*.

## DISCUSSION

In this work, we developed a mathematical model of microdomain formation in the canalicular membrane of hepatocytes and the solubilization of bile micelles from Ld microdomains. Our aim was to check how the lipid and protein composition of the canalicular membrane might control the biliary secretion rate of PC and CH and whether variations in the lipid and protein content of the membrane might account for a diverse set of seemingly contradictory experimental findings.

### Membrane proteins restrain the size of lipid domains

Segregation of membrane lipids into domains differing in lipid composition, ordering state of lipids and preference for specific membrane proteins has been implicated in the organization and sorting of functional protein complexes, formation of connections between the membrane and the cytoskeleton and budding of membrane fragments in endocytosis and exocytosis (Laude and Prior, 2004). With respect to the canalicular membrane of hepatocytes, a specific function of coexisting Lo and Ld microdomains is enabling the extraction of membrane fragments without endangering membrane integrity. With the aim to better understand the molecular mechanisms governing the size, shape and distribution of microdomains, we have extended our previous mathematical model of domain formation (Eckstein et al., 2015) by including the interaction of lipids with membrane proteins. The main finding of model simulations is that the presence of membrane proteins with differing preferences for specific lipid species prevents the complete segregation of lipids into the large ‘macrodomains’ that are typically observed in artificial lipid membranes. The protein:lipid ratio of the membrane determines the average domain size and hence the size of membrane patches extracted into the bile. Whereas a domain-inducing effect of proteins has been already addressed in several experimental studies (reviewed in Jacobson et al., 2007) and also by model simulations (Hinderliter et al., 2001), this is the first report demonstrating, by means of a carefully validated membrane model, that proteins –irrespective of their mode of motion in the membrane – are capable of restraining the formation of lipid domains to the nanoscale size range. Hence, besides the specific functions of the numerous membrane proteins in the structural organization and secretory function of the canalicular membrane, the mere presence of membrane proteins appears to have a significant impact on the structure and size of microdomains and thereby on the lipid secretion rate (Eckstein et al., 2015). Depletion of the protein density of the plasma membrane, for example, owing to enhanced protein internalization at reduced cellular ATP level, as reported for several epithelial cell membranes (Doctor et al., 2000; Mandel et al., 1994), should render the membrane more vulnerable to BS-dependent destruction and accelerate the cytotoxic effect of BS. Another example for the protective role of membrane proteins against potentially toxic bile acids may be the decreased resistance of the hepato-canalicular membrane to hydrophobic bile salts observed in progressive familial intrahepatic cholestasis type 1 (PFIC1), which is caused by a deficiency of the phospholipid flippase ATP8B1 (Paulusma et al., 2006). The liver of PFIC1 mice lack immunohistochemically demonstrable canalicular ectoenzymes. In the light of our model findings, this protein depletion of the outer leaflet may substantially contribute to the reduced membrane resistance against BS, as well as the phospholipid membrane asymmetry accompanying the gene defect.

### The mechanism of BS-dependent biliary lipid extraction

**Fig. 7.**
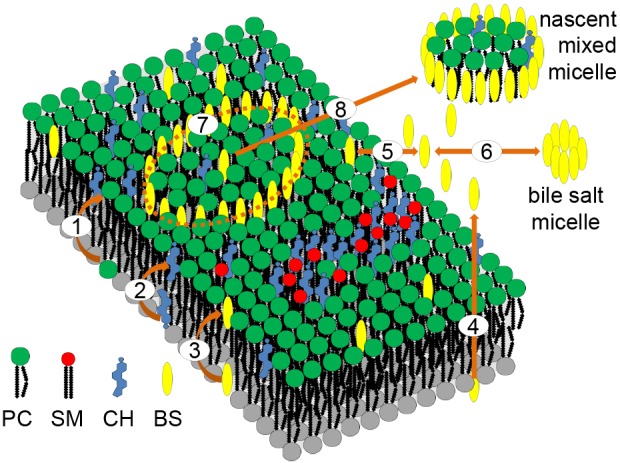
**Schematic of the proposed membrane budding model (B-model) of biliary lipid secretion.** (1) Trans-bilayer movement of PC from the inner to the outer leaflet mediated by MDR3. (2) Trans-bilayer movement of CH from the inner to the outer leaflet mediated by Abcg5–Abcg8. (3) Trans-bilayer flip-flop of BS between the inner and outer leaflet. (4) Transport of BS by BSEP probably operating as a ‘liftase’ presenting CH to luminal BS. (5) Reversible partition of BS monomers between the aqueous lumen and Ld microdomains of the membrane. (6) Formation of BS micelles if the critical micellar concentration of the BS is reached. (7) Arrangement of BS monomers in ring-like structures facilitating membrane budding. (8) Membrane release of disk-like nascent micelles (Hjelm et al., 1992) wrapped by a layer of BS molecules.

In concordance with the general biophysical principles of detergent-dependent membrane solubilization (Helenius and Simons, 1975; Lichtenberg et al., 2013), we postulated a lipid secretion mechanism (B-model) according to which BSs may accumulate in Ld domains in a saturating manner. If the BS concentration in a membrane patch is sufficiently high enough to form circular structures that shield the rim of the patch, extraction of the patch may occur (see Fig. 7). The assumption of such an ‘encircle and cut out’ mechanism of BS-triggered patch release is further backed up by observations showing that polymeric detergents as well as bile acids may form pore-like structures in the membrane (Mazer et al., 1984; Schubert and Schmidt, 1988). For the BS taurocholate, the best agreement between the measured and simulated lipid extraction rates was obtained with setting *K*_BS_=*k*_+_/*k*_−_=5 · 10^−4^ and *η*=1, that is, one lipid molecule is ‘consumed’ to dissolve one BS molecule in the Ld phase of the membrane. Of note, these two model parameters are BS specific.

According to our model simulations, the maximal lipid secretion rates shown in Fig. 3 are accounted for by the saturation of Ld domains with the BS taurocholate. From this it follows that the capacity of lipid transporters and of the intracellular transport of lipids and proteins to the apical membrane is not a limiting factor for TC-dependent lipid extraction as long as the size of Ld microdomains can be kept at an average size of 20–40 lipids and the total leaflet area occupied by Ld domains is not larger than 50%. However, a shift of the canalicular BS composition towards BSs with a higher membrane solubility (i.e. lower values of the solubility index *η* and/or larger values of the partition coefficient *K*_BS_) may give rise to a significant left- or right-shift of the lipid secretion curves (see Fig. 6B) and thus may lead to an imbalance between the membrane-replenishing capacity of the hepatocyte and the elevated extraction of membrane patches. Along this line of argumentation, the differences in the absolute values of the lipid secretion rates obtained in the two independent experiments shown in Fig. 3 may at least be partially due to variations in the BS composition of the bile actually present in the canalicular lumen. The infused TC is taken up by the hepatocyte and can be chemically modified into different BS species having a higher or lower hydrophobic index than TC.

Of note, the half-saturating TC secretion rate of 150 nmol/min/100 g and 600 nmol/min/100 g obtained for the wild-type mice (see Fig. 3) is above the rate that is required to solubilize 8% of the canalicular membrane per minute as estimated by Crawford (Crawford, 1996). Given a liver mass of 3–5% of body weight in mice (Nesteruk et al., 2014), 135×10^6^ hepatocytes per g mice liver (Sohlenius-Sternbeck, 2006) and 218 µm² canalicular membrane surface per hepatocyte (Crawford, 1996), the total area of the apical membrane available for bile formation amounts to 117.72×10^9^ µm²/100 g. Assuming 10^6^ lipids per µm² area of the outer leaflet of the membrane, the lipid secretion rate can be roughly estimated as 16 nmol/min/100 g. With TC as the dominant BS in the experiments shown in Fig. 3, such a secretion rate is achieved with TC secretion rates of 110 nmol/min/100 g in Mdr2^+/−^ mice and 85 nmol/min/100 g in the corresponding wild-type mice or 250 nmol/min/100 g in Abcg8^−/−^ mice and 200 nmol/min/100 g in the corresponding wild-type mice. All these TC secretion rates are below the estimated half-saturation secretion rates.

### The importance of the membrane lipid composition on biliary extraction rates

Experiments in mice with knockdown or overexpression of specific ABC transporters involved in trans-bilayer lipid translocation have provoked a renewed thinking about the mechanism of biliary lipid secretion.

Mice with a disruption of the Mdr2 gene (the mouse homolog of MDR3), were found to be unable to transport PC into bile (Smit et al., 1993). Human MDR3 (ABCB4) mutations result in a wide spectrum of phenotypes, ranging from progressive familial intrahepatic cholestasis type 3 (PFIC3) to adult cholestatic liver disorders (Jacquemin et al., 2001). Intriguingly, our model simulations suggest that a 30% decrease in the lipid secretion rate, as observed in Mdr2 heterozygous mice can be accounted for by a modest PC depletion (from 47% to 37%) of the membrane. Usually the heterozygous genotype with a functional allele and a non-functional allele produces 50% of the standard activity. In this case, the PC content of the membrane should be ∼23% and not 37% as predicted by the model. Hence, there appears to be a compensation for the loss of the non-functional allele, either by enhanced gene expression and anterograde transport of the protein to the membrane and/or reduced retrograde transport from the membrane to the lysosome.

Mutations in human genes encoding for ABCG5 or ABCG8 have been demonstrated to cause sitosterolemia, characterized by an accumulation of sterols in blood and tissues, consequent to the enhanced intestinal absorption and decreased biliary removal of CH and plant sterols (Berge et al., 2000). Yu et al. (2002a) have found that the CH molar ratio in the bile was significantly lower in the Abcg5–Abcg8^−/−^ mice (0.37%) than in the littermate controls (2.2%) whereas the PC content of the bile was not significantly altered. Conversely, overexpression of Abcg5–Abcg8 in mice was accompanied by a more than fivefold increase of biliary CH (Yu et al., 2002b). The outcome of these experiments can be well reproduced by our model if we make the following assumptions: (1) that similar to other trans-bilayer lipid transporters, Abcg5–Accg8 works primarily as a flippase translocating CH from the inner to the outer leaflet. Indeed, in the Abcg5–Abcg8-deficient mice, the CH content of the membrane was found to be reduced by ∼50% (Kosters et al., 2006) suggesting that changes of the Abcg5–Accg8 activity are accompanied by analogous changes of the CH content in the membrane. (2) That changes of the membrane CH are counterbalanced by inverse changes of the SM content. In our three-lipid model, this condition appears to be indispensable in order to preserve the microdomain architecture of the outer leaflet and hence the functional and structural integrity of the membrane. Replacement of CH by SM preserves the size distribution of Lo and Ld domains because increasing SM stabilizes Lo microdomains by dragging more CH from the Ld into the Lo phase. Owing to this redistribution of CH, the Ld phase becomes depleted in CH and accordingly the released micelles have a lower relative CH content.

### Arguments in favor of the budding model

There are several aspects conferring a high degree of credibility to our proposed membrane budding model (B-model) of biliary lipid secretion (Fig. 7). First, the model simulations recapitulate a large array of reported data on the lipid composition of the bile and BS-dependent lipid secretion rates under normal conditions and perturbation of trans-bilayer lipid transport. Second, the model is built on numerous experimental findings on the formation of microdomains obtained in artificial membrane systems and on top takes into account the interaction of lipids and proteins as a crucial domain-stabilizing factor in real biological membranes. Third, the disproportional appearance of PC and CH in the bile observed under extreme situations (CH overload, knock-down or overexpression of lipid translocase) is explained by corresponding variations in the lipid composition of Ld microdomains. In our simulations, the CH:PC ratio in the Ld microdomain varied between 0.01 and 0.39. A maximal solubility limit of up to 66% has been determined for PC bilayers (Huang et al., 1999). Fourth, the secretion mechanism obeys the general biophysical principles governing the detergent-mediated solubilization of biological membranes in general (Helenius and Simons, 1975; Lichtenberg et al., 2005). In particular, the controlled loss of membrane fragments in the process of bile secretion and the disproportional membrane-damaging loss of membrane fragments at critically high BS concentrations can be understood as two facets of one and the same basic mechanism. The high sensitivity of the patch extraction rate against changes in the protein content of the membrane (see Fig. 6A) has to be seen in the context of BS-dependent membrane damage. If the BS concentration reaches a critical threshold where the extraction of membrane proteins exceeds their replenishment by intra-cellular vesicular transport, the protein:lipid ratio of the membrane is decreasing. This may invoke a self-amplifying circle as the lowering of the protein:lipid ratio increases the patch extraction rate. Several studies (Billington et al., 1980; Holdsworth and Coleman, 1976) have reported that a protein loss of ∼20% precedes BS-dependent membrane lysis. According to our model, a protein depletion of that size would result in an about 30-fold increase of the membrane extraction rate which may define the upper limit for the increase of intracellular processes involved in membrane re-filling. In addition, the model may also explain the paradoxical finding that inhibition of the bile salt export pump (BSEP; also known as ABCB11) results in an even higher secretion rate of CH and PC in BSEP^−/−^ mice (Wang et al., 2001). Bile acids may intercalate into the outer leaflet not only from the luminal side but also through translocation from the inner leaflet (Donovan and Jackson, 1997) (process number 3 in Fig. 7). This represents a special secretion mode that works without luminal presence of BS in micellar concentrations as required for CH secretion according to the E-model (Vrins et al., 2007). In response to the blocked BS transporter, BSEP^−/−^ mice upregulate the production of tetra-hydroxy BSs, which owing to their very high hydrophilicity should be able to maintain a secretion rate comparable with that of the wild-type mice. Changing in our model the parameters K_BS_ and η determining the solubility of BS in the Ld phase has a high impact on the secretion rate. Increasing *K*_BS_ from *K*_BS_=2.5 · 10^−4^ to *K*_BS_=10^−3^ shifts the half-saturation constant from 220 down to 60 nmol/min/100 g, and decreasing the parameter *η* from *η*=1 to *η*=3/4 raises the saturation level of the secretion by 30% (see Fig. 6B). These changes together may elicit a 6-fold higher solubilization efficiency. Finally, budding from the external hemi-leaflet provides an explanation of how luminal BSs can extract large quantities of phospholipid on the basis of their detergent action, without disrupting the integrity of the detergent-resistant canalicular plasma membrane.
